# Response to Letter to the Editor, “Mechanical Ventilation in COVID-19 Patients: a question from Age to Frailty”

**DOI:** 10.14336/AD.2022.0819-1

**Published:** 2023-04-01

**Authors:** Fiona Ecarnot, Paola Rebora, Giuseppe Bellelli, for the FRACoViD Team

**Affiliations:** ^1^EA3920, University of Franche-Comté and Department of Cardiology, University Hospital Besançon, Besançon, France.; ^2^Bicocca Center of Bioinformatics, Biostatistics and Bioimaging, School of Medicine and Surgery, University of Milano-Bicocca, Monza, Italy.; ^3^School of Medicine and Surgery, University of Milano - Bicocca, Italy.; ^4^Acute Geriatric Unit, San Gerardo hospital, Monza, Italy.

## To Editor,

We welcome the letter by Dr Esquinas and colleagues in response to our paper on the role of age and frailty in selecting patients eligible for mechanical ventilation during the first two waves of COVID-19 pandemic [1]. The authors raised three points to be clarified or discussed, and we appreciate the opportunity to provide additional insights.

The first point concerns the hypothesis that in our population, patients needing intubation and mechanical ventilation may have had greater oxygen requirements at admission in comparison to the others. We evaluated the level of O2 saturation (sat O2) on admission in all patients. There was no significant difference in oxygen saturation at admission between those requiring mechanical ventilation and those not requiring mechanical ventilation as the highest level of respiratory support in the multivariable analysis. This suggests that oxygen requirement was not a key element in deciding whether to intubate and support with mechanical ventilation or not in the study cohort.

The second point regards the ROX index. Unfortunately, we did not compute the ROX index in the database, which is a limitation of the study. We thank the authors for pointing out the promising findings with the use of this metric to predict treatment failure in COVID-19 patients receiving continuous positive airway pressure.

The third point focuses on the assessment of frailty in our patients. We agree with Dr Esquinas and colleagues that frailty is a better predictor of outcome in COVID-19 patients than chronological age. This has been demonstrated by several papers in both COVID-19 and non-COVID-19 patients [2-4]. However, we disagree that frailty was prevalent in as many as 45% of patients in our cohort. We also take issue with the hypothesis that if the sample size had included a greater number of older patients, the association between frailty and mechanical ventilation might have become apparent. The Frailty Index (FI) is a method developed by Rockwood and Mitnitski to assess, in a single variable, a wide range of age-related health deficits (e.g., symptoms, signs, laboratory abnormalities) and to capture the person’s heterogeneity in health status, which is not better accounted for by chronological age [5]. The FI is conceived as a continuous variable, although there is general agreement that a FI score =0.25 should be considered as the cut-off above which a patient is deemed to be frail. In our population of 1344 patients [2], the median frailty index was 0.088 (0.03, 0.20), indirectly suggesting that only a minority of our cohort was actually frail. It is also important to highlight that, using a threshold of FI score=0.13, which was previously shown to be associated with mortality in our study cohort, there were differences in the rates of use of several treatments in unadjusted analysis, including antibiotics, hydroxychloroquine, antivirals (Lopinavir/rotinavir, Darunavir, Remdesivir) and tocilizumab. There was no significant difference in the use of steroids between those with a FI <0.13 and those with a FI above this threshold. All the other treatments were less frequently prescribed in those with a higher FI, with the exception of antibiotics, which were more frequently prescribed in the frailer patients. It is thus unlikely that the treatments could account for the lower rate of mechanical ventilation in frailer patients.

In conclusion, we firmly believe that accurate frailty assessment would have made it possible to identify the patients in our cohort who were at greatest risk of negative outcomes. In this way, appropriate ventilatory support and mechanical ventilation could have been offered to people who, instead, may have been denied these treatments solely on the basis of age. Our hope is to enhance understanding and improve knowledge of frailty among intensivists and non-geriatric specialists in hospitals caring for older people.
